# Large-scale RNA-Seq Transcriptome Analysis of 4043 Cancers and 548 Normal Tissue Controls across 12 TCGA Cancer Types

**DOI:** 10.1038/srep13413

**Published:** 2015-08-21

**Authors:** Li Peng, Xiu Wu Bian, Di Kang Li, Chuan Xu, Guang Ming Wang, Qing You Xia, Qing Xiong

**Affiliations:** 1 State Key Laboratory of Silkworm Genome Biology, Southwest University, Chongqing 400715, China; 2 Institute of Pathology and Southwest Cancer Center, Southwest Hospital, Third Military Medical University, Chongqing 400038, China; 3 Department of Computer Science and Technology, Department of Statistics, Southwest University, Chongqing 400715, China; 4 Department of Oncology, Chengdu Military General Hospital of PLA, Chengdu 610083, China; 5 Department of Pathology, Clinical School, Dali University, Dali 671000, China

## Abstract

The Cancer Genome Atlas (TCGA) has accrued RNA-Seq-based transcriptome data for more than 4000 cancer tissue samples across 12 cancer types, translating these data into biological insights remains a major challenge. We analyzed and compared the transcriptomes of 4043 cancer and 548 normal tissue samples from 21 TCGA cancer types, and created a comprehensive catalog of gene expression alterations for each cancer type. By clustering genes into co-regulated gene sets, we identified seven cross-cancer gene signatures altered across a diverse panel of primary human cancer samples. A 14-gene signature extracted from these seven cross-cancer gene signatures precisely differentiated between cancerous and normal samples, the predictive accuracy of leave-one-out cross-validation (LOOCV) were 92.04%, 96.23%, 91.76%, 90.05%, 88.17%, 94.29%, and 99.10% for BLCA, BRCA, COAD, HNSC, LIHC, LUAD, and LUSC, respectively. A lung cancer-specific gene signature, containing SFTPA1 and SFTPA2 genes, accurately distinguished lung cancer from other cancer samples, the predictive accuracy of LOOCV for TCGA and GSE5364 data were 95.68% and 100%, respectively. These gene signatures provide rich insights into the transcriptional programs that trigger tumorigenesis and metastasis, and many genes in the signature gene panels may be of significant value to the diagnosis and treatment of cancer.

Recent advances in cancer genomics have created a rich resource for studying the causes of cancer. The Cancer Genome Atlas (TCGA)^1^ (http://cancergenome.nih.gov) has accrued more than 10,000 cases of human cancer including over 25 different cancer types. Datasets including RNA-Seq, miRNA-Seq, Exon-Seq, somatic mutations, methylation, CNV for each case are publically available via the TCGA Data Portal (https://tcga-data.nci.nih.gov/tcga/tcgaHome2.jsp) and UCSC Cancer Genomics Hub (https://cghub.ucsc.edu). Translating these data into biological insights remains a major challenge. Currently several studies have analyzed genome-wide mutational patterns in different cancer types and identified genes harboring functional mutations implicated in cancerogenesis^2^,^3^,^4^,^5^. Cancer is thought to be driven by gene expression pattern changes due to the accumulation of mutations or epigenetic modifications; thus, a comprehensive characterization of alterations in gene expression will not only advance our understanding of cancer biology, it will also provide a large number of new potential diagnostic and therapeutic targets for cancer. Cheng *et al.*^6^ introduced a method to identify cancer-associated attractors and revealed some interesting bimolecular events shared among multiple cancer types based on microarray gene expression data. However, genome-wide association analysis of RNA-Seq transcriptome data across various TCGA cancer types has rarely been reported. RNA-Seq, a revolutionary technology for genome-wide gene expression profiling, offers several key advantages compared to microarrays^7^, it could better characterize the transcriptomic changes associated with human cancers.

In this study, we analyzed and compared the RNA-Seq transcriptomes of 4043 cancer and 548 solid tissue normal samples across 21 types of cancer from TCGA. We created a catalog of gene expression alterations for each cancer type, and our results show that the alterations in gene expression vary substantially between different tumor types. Studies have shown that cancer involves many different genes and a majority of these genes have a small to moderate effect^8^, it is difficult to detect these effects by single gene analysis. By clustering genes into co-regulated gene sets, we are able to examine accumulative effects of a group of functionally related genes. We performed gene set association analysis for each cancer type; our results revealed several common gene signatures shared by multiple cancer types and a lung cancer-specific gene signature. We also validated these signatures using several non-TCGA data sets. These cross-cancer and cancer-specific transcriptional aberrations improve our understanding of the etiology of human cancers, and are of great importance for the diagnosis and treatment of cancer.

## Results

### Gene-level differential expression analysis of transcriptomes

We conducted gene differential expression analysis and created a catalog of gene expression alterations for each of 12 cancer types; the results are shown in Supplementary Table S1. Our results show that a large number of genes were differentially expressed. Among a total of 20530 genes, the percentage of differentially expressed (DE) genes with FDR < 0.01 is 0.32, 0.72, 0.51, 0.52, 0.52, 0.65, 0.68, 0.54, 0.69, 0.46, 0.46, and 0.56 for BLCA, BRCA, COAD, HNSC, LIHC, LUAD, LUSC, KICH, KIRC, KIRP, PRAD, and THCA, respectively. To examine the similarity of DE genes between cancer types, we extracted the top 3% most differentially expressed genes from each cancer type. We then calculated the number of common DE genes between cancer types. As shown in Table 1, we found that DE genes vary substantially across cancer types, and there are less than 20% common DE genes between most cancer types. LUAD and LUSC, two forms of lung cancers, turn out to be most similar cancers since they share 55% of DE genes. Contrarily, the DE profiles of two kidney cancers, KICH and KIRC, are quite different from each other and others; the percentage of common DE genes is less than 10%. Additionally, THCA is also poorly overlapped with other cancers in terms of DE genes. The diversity in differential expression could be explained by several factors: (1) many of gene expression alterations may be cancer type-specific; (2) aberrations in different genes may have same phenotypic consequences; (3) single gene analysis may miss many subtle effects on causative genes.

### Gene clustering

Prior to gene set association analysis, we clustered genes based on their expression profiles over all normal samples across 12 cancer types. We obtained a total of 3236 clusters (Supplementary Table S2). The expression changes of genes in a cluster are highly correlated under various conditions, thus, it is reasonable to assume that genes in the same cluster are co-regulated or belong to the same pathway.

### Gene set association analysis of TCGA data

Cancers arise from the aberrations in multiple genes, many of which only have moderate or weak effect sizes that are difficult to detect by only analyzing individual genes, therefore, we adopted gene set association analysis to detect the accumulative effect of a group of functionally related genes and to reveal the transcriptional program accounting for the variability in phenotype.

Carcinogenesis is caused by the accumulation of mutations and epimutations in normal cells^9^,^10^, which confer a growth and selective advantage upon these cells, resulting in uncontrolled cell division and the evolution of these cells by natural selection^11^. The mutations can be classified into two classes, driver mutation and passenger mutation, according to their phenotypic effects^12^. Driver mutations are causally implicated in carcinogenesis while passenger mutations don’t contribute to the development of cancer. A driver mutation is expected to alter the gene expression of its target genes and/or genes that share the same biological pathway^13^,^14^, and these changes in gene expression account for the phenotypic variance^15^.

**Figure 1:**
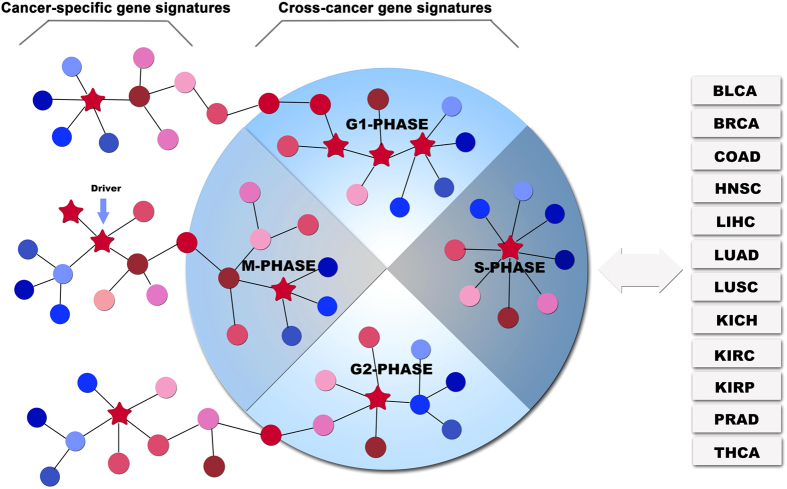
Two possible carcinogenic mechanisms. (1) gene expression aberrations in cell cycle-associated pathways can directly lead to carcinogenesis, these pathways are cross-cancer gene signatures altered across a range of cancer types; (2) gene expression aberrations in organ-specific pathways can indirectly lead to carcinogenesis by interacting with cell cycle-associated pathways, these pathways are cancer-specific gene signatures altered in a single cancer type. Stars represent driver mutations that can alter the expression levels of their target genes.

The cell cycle lies at the core of cancer^16^,^17^. In normal cells, the cell cycle is controlled by a series of signaling pathways by which a cell grows, replicates its DNA and divides. In cancers, as a result of mutations, this regulatory process malfunctions, resulting in uncontrolled cell proliferation that leads to carcinogenesis^18^,^19^. From the perspective of pathway, we hypothesize that there may be two potential carcinogenic mechanisms, as illustrated in Fig. 1: (1) one or more driver mutations are within a cell cycle-associated pathway, altering its expression pattern and consequently leading to cancer; (2) one or more driver mutations lie in an organ/tissue-specific pathway or other pathways not related to cell cycle, which interacts with a cell cycle-associated pathway, alters its expression pattern, and ultimately results in cancer. Since the deregulation of cell cycle is a common characteristic shared by multiple cancer types, we expected that the expression of cell cycle-associated pathways would be altered across a range of cancers. By analyzing and comparing the transcriptome data of 12 cancer types, we can test this hypothesis.

**Figure 2:**
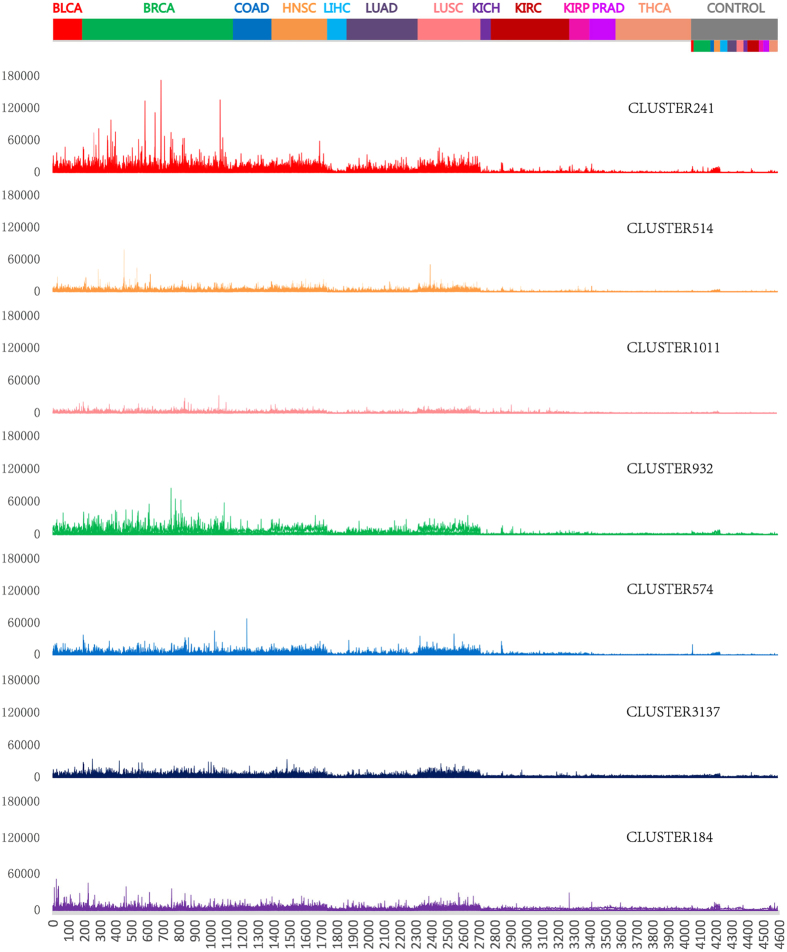
The normalized expression levels of seven cross-cancer gene signatures across 12 types of cancer and normal samples.

A gene signature denotes a set of genes that are significantly differentially expressed between cancer and normal samples. We call those pathways/gene sets significantly altered in multiple cancer types as cross-cancer gene signatures while those disrupted in just one cancer type as cancer-specific gene signatures. We performed gene set association analysis using all gene sets generated by gene clustering; the results are shown in Supplementary Table S3. We identified 20, 7, 7, 6, 7, 15, 30, and 1 significant gene sets for BLCA, BRCA, COAD, HNSC, LIHC, LUAD, LUSC, and KICH, respectively. No significant associations were found for KIRC, KIRP, PRAD, and THCA. Among 46 significant gene sets, seven are cross-cancer gene signatures whose expression levels were significantly altered in at least four cancer types (Fig. 2), the false discovery rates (FDRs) of these gene sets for each cancer type are shown in Table 2. In order to gain biological insights into these gene sets, we performed three types of pathway enrichment analyses, GO analysis, KEGG analysis, and Pathway Commons analysis, and disease association analysis for genes of each of these gene sets. The results of these analyses are shown in Supplementary Table S4. Interestingly, we found that these seven cross-cancer gene signatures are all closely related to cell cycle regulation, as we expected. Gene set CLUSTER2556 is significant in BLCA, COAD, and LUSC. There are 9 significant gene sets shared by two cancer types. Gene set CLUSTER242 is shared by LIHC and LUSC, and the remaining 8 gene sets are shared by LUAD and LUSC. LUAD and LUSC are more similar to one another than other cancer types possibly because they are both lung cancers.

#### Cross-cancer gene signatures

We identified seven cross-cancer gene signatures: CLUSTER241, CLUSTER514, CLUSTER1011, CLUSTER932, CLUSTER574, CLUSTER3137, and CLUSTER184, that were altered in at least four types of human cancers. All of these signatures are associated with cell cycle regulation.

##### Cross-cancer gene signature 1 – CLUSTER241.

CLUSTER241 is significantly altered in seven cancer types: BLCA, BRCA, COAD, HNSC, LIHC, LUAD, and LUSC. GO analysis, KEGG analysis, and Pathway Commons analysis indicate that genes in this cluster are enriched in pathways involved in the cell cycle. The top enriched GO biological process, KEGG pathway, and Pathway Commons pathway are M Phase, Cell Cycle, and Mitotic Prometaphase, respectively. The top associated disease is Aneuploidy. Aneuploidy, denoting cells with an abnormal number of chromosomes, is commonly observed in human cancer; it has been recognized as a key characteristic of cancer^20^,^21^. This cluster contains 33 genes, several of which have reported roles in cancer. Kinesins have been reported to play critical roles in the initiation and development of human cancers^22^,^23^. Marker of proliferation Ki-67 (*MKI67*) is a prognostic marker for breast cancer^24^,^25^. Simultaneous aberration of topoisomerase (DNA) II alpha (*TOP2A*) and v-erb-b2 avian erythroblastic leukemia viral oncogene homolog 2 (*ERBB2*/*HER2*) has been observed in multiple tumor types^26^,^27^.

##### Cross-cancer gene signature 2 – CLUSTER514.

CLUSTER514 is significantly altered in seven cancer types: BLCA, BRCA, COAD, HNSC, LIHC, LUAD, and LUSC. GO analysis, KEGG analysis, and Pathway Commons analysis indicate that genes in this cluster are enriched in pathways involved in the cell cycle. The top enriched GO biological process, KEGG pathway, and Pathway Commons pathway are Organelle Fission, Cell Cycle, and Cell Cycle, Mitotic, respectively. The top associated disease is Cancer or Viral Infections. This cluster contains 36 genes, of which a lot are prognostic markers for cancer. Enhancer of zeste 2 polycomb repressive complex 2 subunit (*EZH2*) has been linked to multiple cancers^28^,^29^. Aurora kinase A (*AURKA*) causes chromosome instability by inactivating p53 and contributes to tumorigenesis/carcinogenesis^30^,^31^,^32^. Baculoviral IAP repeat containing 5 (*BIRC5*) is over-expressed in most human cancers; the microRNA targeting BIRC5 suppresses cell proliferation in triple-negative breast cancer (TNBC) cells^33^,^34^,^35^. Thymidine Kinase 1 (*TK1*), which is elevated in the early stages of malignancies, is a universal marker for cancer^36^,^37^. Polo-like kinase 1 (*PLK1*) is overexpressed in many tumor types; it is a target for cancer therapy^38^,^39^,^40^. RAD51 recombinase (*RAD51*) plays a critical role in DNA Damage Repair and is a potential therapeutic target for cancer^41^,^42^. Hyaluronan-mediated motility receptor (*HMMR*) is correlated to the stemness and tumorigenicity of cancer stem cells^43^,^44^. Cyclin B1 (*CCNB1*), PDZ binding kinase (*PBK*), and cyclin-dependent kinase inhibitor 3 (*CDKN3*) are also prognostic biomarkers for various types of cancer^45^,^46^,^47^,^48^,^49^.

##### Cross-cancer gene signature 3 – CLUSTER1011.

CLUSTER1011 is significantly altered in seven cancer types: BLCA, BRCA, COAD, HNSC, LIHC, LUAD, and LUSC. GO analysis, KEGG analysis, and Pathway Commons analysis indicate that genes in this cluster are enriched in pathways involved in the cell cycle. The top enriched GO biological process, KEGG pathway, and Pathway Commons pathway are Cell Cycle, Cell Cycle, and DNA Replication, respectively. The top associated disease is Fanconi Anemia (FA). The FA proteins are involved in the cell-cycle checkpoint and DNA-repair pathways^50^,^51^. This cluster contains 19 genes, several of which have been linked to cancer. Mutations in BRCA1 interacting protein C-terminal helicase 1 (*BRIP1*) have been associated with ovarian cancer and breast cancer^52^,^53^,^54^. The overexpression of *KIAA1524*/*CIP2A* have been observed in multiple types of cancer^55^,^56^,^57^. Centromere protein H (*CENPH*) is a prognostic marker for cancer^58^,^59^,^60^,^61^.

##### Cross-cancer gene signature 4 – CLUSTER932.

CLUSTER932 is significantly altered in seven cancer types: BLCA, BRCA, COAD, HNSC, LIHC, LUAD, and LUSC. GO analysis, KEGG analysis, and Pathway Commons analysis indicate that genes in this cluster are enriched in pathways involved in the cell cycle. The top enriched GO biological process, KEGG pathway, and Pathway Commons pathway are Cell Cycle, Cell Cycle, and Cell Cycle, Mitotic, respectively. The top associated disease is Retinoblastoma. This cluster contains 19 genes, many of which have well-known roles in cancer. Cyclin E1 (*CCNE1*) and cyclin E2 (*CCNE2*) play critical roles in cell cycle regulation and are potential therapeutic targets in cancer^62^,^63^,^64^. The aberrant expression of cell division cycle 6 (*CDC6*) has been documented in multiple human cancers^65^,^66^,^67^. E2F transcription factor 7 (*E2F7*) is interacted with p53, it has been implicated as playing a role in tumorigenesis^68^,^69^,^70^. Ubiquitin-like with PHD and ring finger domains 1 (*UHRF1*) is an upstream regulator of the Tip60-p53 interaction and it has been linked to liver cancer^71^.

##### Cross-cancer gene signature 5 – CLUSTER574.

CLUSTER574 is significantly altered in six cancer types: BRCA, COAD, HNSC, LIHC, LUAD, and LUSC. GO analysis, KEGG analysis, and Pathway Commons analysis indicate that genes in this cluster are enriched in pathways involved in the cell cycle. The top enriched GO biological process, KEGG pathway, and Pathway Commons pathway are Mitotic Cell Cycle, Pyrimidine Metabolism, and Cell Cycle, Mitotic, respectively. The top associated disease is Pancreatic Diseases. This cluster contains 17 genes, several of which have been associated with cancer. Forkhead box M1 (*FOXM1*) is overexpressed in the majority of human cancers, it has well-known roles in cancer^72^,^73^,^74^. Thymidylate synthetase (*TYMS*) is considered a prognostic biomarker for cancer^75^,^76^. Ribonucleotide reductase M2 (*RRM2*) is associated with poor survival; it is also implicated in angiogenesis^77^,^78^,^79^. Spindle and kinetochore associated complex subunit 1 (*SKA1*) has been highlighted as a biomarker in several types of cancers^80^.

##### Cross-cancer gene signature 6 – CLUSTER3137.

CLUSTER3137 is significantly altered in five cancer types: BLCA, COAD, LIHC, LUAD, and LUSC. GO analysis, KEGG analysis, and Pathway Commons analysis indicate that genes in this cluster are enriched in pathways involved in the cell cycle. The top enriched GO biological process, KEGG pathway, and Pathway Commons pathway are Cell Cycle Process, Cell Cycle, and Mitotic M-M/G1 Phases, respectively. The top associated disease is Retinoblastoma. This cluster contains 16 genes, several of which have been linked to cancer. S-phase kinase-associated protein 2, E3 ubiquitin protein ligase (*SKP2*) is a protooncogene in human tumors and is a potential cancer drug target^81^,^82^,^83^. Ribonucleotide reductase M1 (*RRM1*) is a prognostic marker for cancer^84^,^85^. DNA (cytosine-5-)-methyltransferase 1 (*DNMT1*) is overexpressed in many cancers and is correlated to the aberrant methylation in human cancer cells^86^. The polymorphisms of *DNMT1* have been reported to increase breast cancer risk^87^,^88^,^89^.

##### Cross-cancer gene signature 7 – CLUSTER184.

CLUSTER184 is significantly altered in four cancer types: BLCA, BRCA, LUAD, and LUSC. GO analysis, KEGG analysis, and Pathway Commons analysis indicate that genes in this cluster are enriched in pathways involved in the cell cycle. The top enriched GO biological process, KEGG pathway, and Pathway Commons pathway are M Phase, Oocyte Meiosis, and Cell Cycle, Mitotic, respectively. The top associated disease is Aneuploidy. This cluster contains 24 genes, several of which have been linked to cancer. The aurora kinase B (*AURKB*) was shown to be overexpressed in many types of cancer cells, and it has been implicated in the carcinogenesis and tumor development process^90^,^91^,^92^. Ubiquitin-conjugating enzyme E2C (*UBE2C*/*UBCH10*) has been reported to play a critical role in carcinogenesis and tumor development^93^,^94^,^95^.

Although these seven cross-cancer gene signatures in a broad sense are involved in the cell cycle, they may manifest different cellular processes leading to the abnormal cell cycle regulation in malignancy. For example, DNMT1 in CLUSTER3137 is the major enzyme responsible for maintenance of the DNA methylation pattern^96^,^97^,^98^. *DNMT1* has been reported to be overexpressed in many cancers and to be involved in the epigenetic silencing of tumor suppressor genes in human tumor cells^86^. Therefore, the perturbation of CLUSTER3137 might be an epigenetic trigger of tumorigenesis. The deregulation of CLUSTER1011 may reveal the roles of components of the Fanconi anemia/BRCA pathway in human cancers. Increasing evidence shows that FA proteins are involved in the DNA damage response^50^,^51^. In this cluster, except for genes that have established roles in the DNA damage response, such as Fanconi anemia, complementation group D2 (*FANCD2*)^99^, our study also suggests genes, e.g., downstream neighbor of SON (DONSON) and proline/serine-rich coiled-coil 1 (PSRC1), that may have new unrevealed functions in DNA repair since the expression levels of theses genes were up-regulated in accordance with FA proteins and BRIP1 in cancer samples. Altogether, these seven cross-cancer gene signatures can not only deepen and broaden our understanding of the cellular events involving carcinogenesis related to the four phases of the cell cycle, they also reveal many potential novel therapeutic targets that have so far not been linked to cancers but may have unknown roles in cancer biology. Our study can be considered as a starting point, and further investigations (e.g., mutation analysis, survival analysis, and functional analysis) on these genes or clusters may lead to the discovery of novel cancer biomarkers and development of new anticancer therapies.

#### Gene signatures significantly altered in one type of cancer

**Figure 3:**
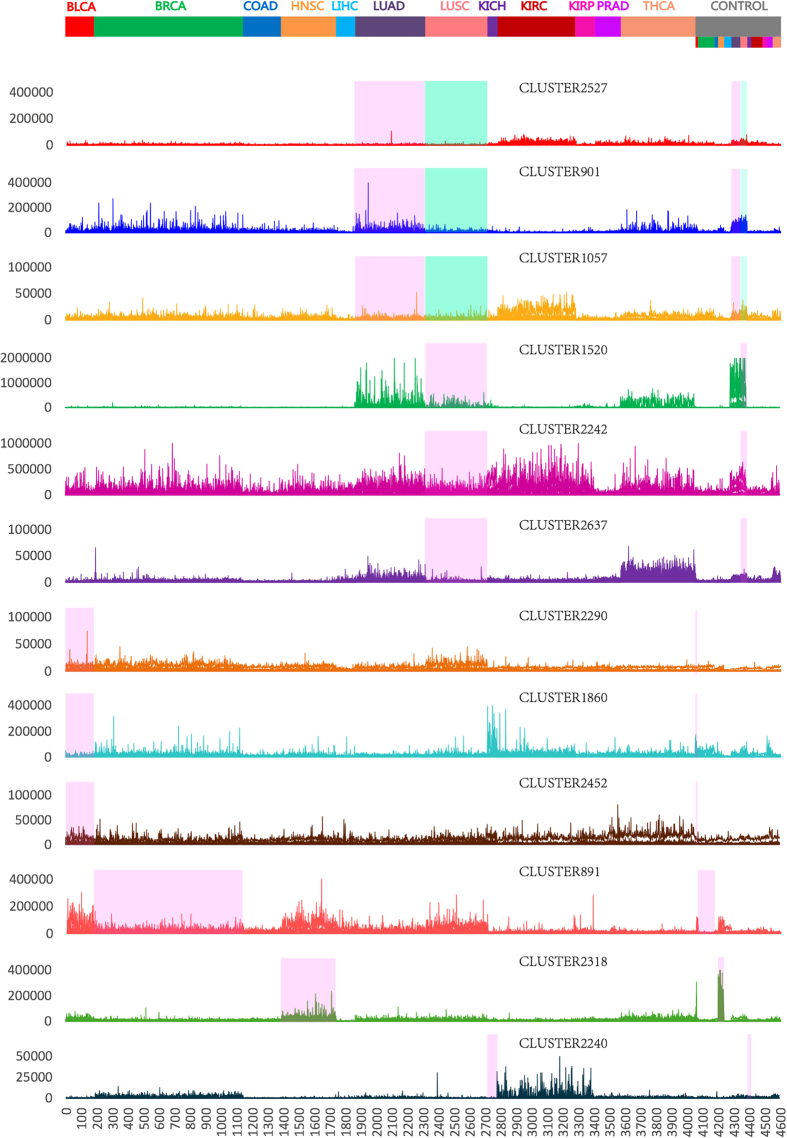
The normalized expression levels of gene signatures significantly altered in one type of cancer across 12 types of cancer and normal samples.

Based on the TCGA cancer data sets we used, we identified 37 gene signatures significantly altered (FDR < 0.15) only in one type of cancer, of which 21 gene signatures are for lung cancers: LUAD and/or LUSC, 13 for BLCA, 1 for BRCA, 1 for HNSC, and 1 for KICH. Figure 3 lists the expression patterns of part of these gene signatures across cancer and normal samples. Among these signature gene sets, several were implicated in relative organ-specific diseases by disease association analysis, and may provide insights into transcriptional aberrations underlying the initiation and progression of a specific cancer type.

##### Gene signatures for lung cancers.

21 clusters were significantly altered only in one or both of lung cancers, three of which, CLUSTER1520, CLUSTER901 and CLUSTER1057, have been implicated in lung diseases.

**Figure 4:**
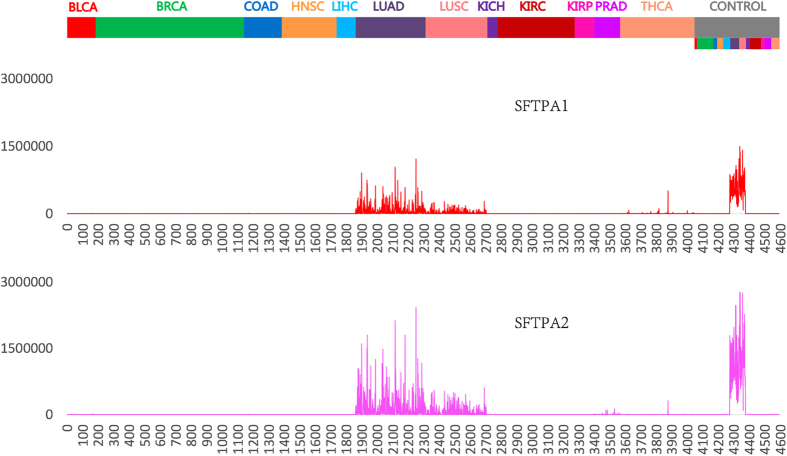
The normalized expression levels of SFTPA1 and SFTPA2 across 12 types of cancer and normal samples.

CLUSTER1520 contains 39 genes. Some genes in this cluster have been reported to be associated with lung cancer or other lung diseases (see Table 3 for details). Among them, two genes, SFTPA1 and SFTPA2, encode surfactant protein A (SP-A) that plays a vital role in maintaining normal lung function^100^ and have been implicated in various lung diseases^101^,^102^,^103^,^104^,^105^,^106^,^107^,^108^,^109^. The expression levels of SFTPA1 and SFTPA2 were much higher in lung tissue samples than in any other tissue samples, moreover, these two genes were strikingly down-regulated in lung tumor tissues as compared to the adjacent nontumor tissues (Fig. 4). We thus speculate that the expression changes in these two genes might be an important indicator for lung function abnormalities, and those 39 genes in CLUSTER1520 might form a network underlying the initiation and/or development of lung cancers. It could be valuable to elucidate the possible roles of these genes in lung cancer in an experimental setting.

The top associated disease for CLUSTER901 is Lung Neoplasms (adjP = 0.0006). This cluster contains 32 genes, several of which have been reported to play roles in lung diseases. G protein-coupled receptor, class C, group 5, member A (*Gprc5a*) protein is detected in the lungs more than in any other tissue; Gprc5a knockout promotes lung inflammation and tumorigenesis in mice^110^,^111^,^112^. Moreover, *GPRC5A* is down-regulated in the adjacent field and normal bronchial epithelia of patients with chronic obstructive pulmonary disease and non-small-cell lung cancer^113^,^114^. Wingless-type MMTV integration site family, member 7A (*WNT7A*) has been reported to be associated with lung cancer^115^,^116^. Claudin 18 (*CLDN18*) deficiency is related to alveolar barrier dysfunction^117^,^118^. Adrenoceptor beta 2 (*ADRB2*) is associated with lung function and lung diseases^119^,^120^.

The top associated diseases for CLUSTER1057 are Lung Diseases (adjP = 0.0037), Respiratory Tract Diseases (adjP = 0.0037), and Airway Obstruction (adjP = 0.0037). CLUSTER1057 contains many immunity-associated genes and might contribute to the immune reactions to lung cancers. Among them, interleukin 33 (*IL33*) has been linked to lung diseases^121^,^122^; interferon (alpha, beta and omega) receptor 2 (IFNAR2) is a prognostic biomarker for lung cancer^123^; GTPase, IMAP family member 6 (GIMAP6) and member 8 (GIMAP8) were significantly down-regulated in the non-small cell lung cancer^124^.

##### Gene signatures for BLCA.

Thirteen clusters were significantly altered only in BLCA, two of which, CLUSTER2174 and CLUSTER1860, have been implicated in bladder abnormalities. The top associated disease for CLUSTER2174 is Urogenital Abnormalities (adjP = 0.0008). This cluster contains 15 genes. Among them, fibroblast growth factor receptor 1 (*FGFR1*) is a well-known gene that plays a key role in the development of urothelial carcinomas^125^,^126^. The top associated disease for CLUSTER1860 is Cystitis (adjP = 3.08e-05). This cluster contains 18 genes. Gap junction protein, gamma 1 (*GJC1*/*CX45*) is one of the two most important gap junction proteins in bladder smooth muscle cells and suburothelial myofibroblasts that are essential for the coordination of normal bladder function^127^. SPARC-like 1 (*SPARCL1*) is down-regulated in bladder cancer and prostate cancer^128^,^129^.

##### Gene signatures for BRCA.

CLUSTER891 is significantly altered only in BRCA. The top associated disease is Adenocarcinoma (adjP = 0.0182). This cluster contains 16 genes, two of which have been linked to p53. p53 represses hepatoma-derived growth factor (*HDGF*), and loss of p53 function contributes to tumorigenesis by elevating *HDGF* expression^130^,^131^. p53 induces the expression of ferredoxin reductase (*FDXR*) which sensitizes cells to apoptosis^132^,^133^. Syndecan 1 (*SDC1*) promotes tumor angiogenesis and growth^134^,^135^.

##### Gene signatures for KICH.

CLUSTER2240 is significantly altered only in KICH. The top associated disease for CLUSTER2240 is Ciliary Motility Disorders (adjP = 1.85e-05), and Ciliary dysfunction is a risk factor for both syndromic and isolated kidney cystic disease^136^. This cluster contains 25 genes. Nephronophthisis 1 (*NPHP1*/*NPH1*) gene deletion is correlated with nephronophthisis^137^,^138^,^139^,^140^.

We have shown that DE genes vary dramatically across 12 cancer types. To test whether there exists a more consistent DE pattern at the gene set level, we extracted the top 3% most differentially expressed gene sets from each cancer type, and calculated the number of common DE gene sets between cancer types. The results are shown in the upper triangular matrix in Table 1. We found that the percentage of common gene sets increase compared to the percentage of common genes for most of cancer pairs. This suggests there are common patterns shared by different tumor types, and these patterns can be detected more effectively at the gene set level. These similarities across cancer types shed light on biomarkers that can be used across a range of cancer types and thus have important implications for treatment.

Through genome-wide gene set association analysis of all co-regulated clusters, we identified both cross-cancer gene signatures, which regulate the cell cycle, and cancer-specific gene signatures, which are associated with relative organ/tissue-specific diseases. These partly verified our hypothesis that alterations in cell cycle-associated pathways directly contribute to the initiation and development of cancers, while some organ/tissue-specific pathways can lead to cancers possibly by altering the expression of cell-cycle associated pathways. More functional investigations are necessary for further validating this hypothesis.

### Leave-one-out cross validation

**Figure 5:**
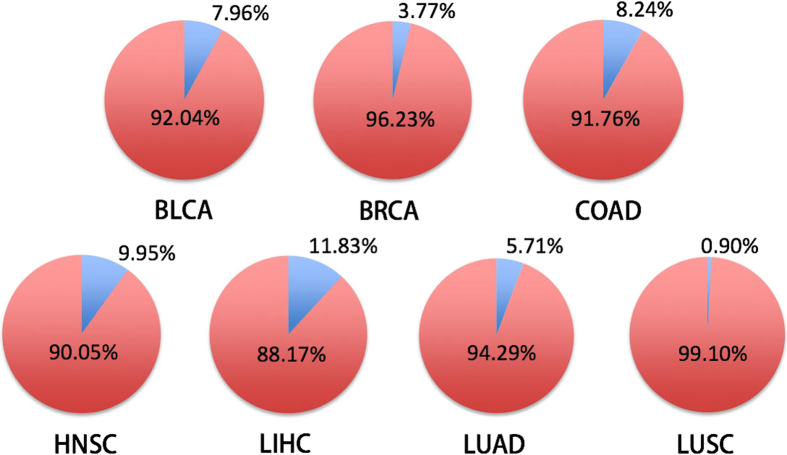
The predictive accuracy and error rates of LOOCV for each cancer type using the 14-gene signature. Red indicates the predictive accuracy; Blue represents error rates.

Seven gene sets, CLUSTER241, CLUSTER514, CLUSTER1011, CLUSTER932, CLUSTER574, CLUSTER3137, and CLUSTER184, were differentially expressed in at least four of the seven cancer types: BLCA, BRCA, COAD, HNSC, LUAD, and LUSC. We extracted the top two most differentially expressed genes from these gene sets and created a 14-gene signature, including kinesin family member 4A (*KIF4A*), nucleolar and spindle associated protein 1 (*NUSAP1*), Holliday junction recognition protein (*HJURP*), NIMA-related kinase 2 (*NEK2*), Fanconi anemia, complementation group I (*FANCI*), denticleless E3 ubiquitin protein ligase homolog (Drosophila) (*DTL*), *UHRF1*, flap structure-specific endonuclease 1 (*FEN1*), IQ motif containing GTPase activating protein 3 (*IQGAP3*), kinesin family member 20A (*KIF20A*), tripartite motif containing 59 (*TRIM59*), centromere protein L (*CENPL*), chromosome 16 open reading frame 59 (*C16orf59*), and *UBE2C*. We employed leave-one-out cross-validation (LOOCV) to assess whether or not this 14-gene signature can be used to differentiate between the normal and cancerous tissue samples of those seven cancer types. Machine learning techniques, for example support vector machines, have been playing a vital role in sample classification^141^,^142^,^143^,^144^. LOOCV was performed using SVM-light^145^ (http://svmlight.joachims.org/) that is an implementation of support vector machines. The predictive accuracy of LOOCV for each cancer type are shown in Fig. 5. The predictive accuracy is the proportion of the total number of predictions that were correct. We found that most of samples were correctly classified based on the expression levels of these 14 genes, the classification accuracy for BLCA, BRCA, COAD, HNSC, LIHC, LUAD, and LUSC were 92.04%, 96.23%, 91.76%, 90.05%, 88.17%, 94.29%, and 99.10%, respectively.

### Validation of the 14-gene cross-cancer signature and a cancer-specific gene signature, CLUSTER1520, on non-TCGA data sets

We have shown that the cancerous and adjacent normal samples from BLCA, BRCA, COAD, HNSC, LIHC, LUAD and LUSC can be precisely classified using the 14-gene cross-cancer signature. To test if the same holds true for other non-TCGA data sources, we downloaded two RNA-Seq data sets, GSE40419^146^ and GSE50760^147^, and one microarray data set, GSE5364^148^, from the Gene Expression Omnibus (GEO: http://www.ncbi.nlm.nih.gov/geo). GSE40419 includes the RNA-Seq expression values for 87 lung adenocarcinomas and 77 adjacent normal tissues, while GSE50760 contains the RNA-Seq expression values of 54 samples (18 primary colorectal cancer, 18 liver metastasis, and 18 normal colon) generated from 18 colorectal cancer patients. We performed LOOCV on these two data sets based on the expression values of the 14-gene cross-cancer signature. We found that the tumor and normal samples were accurately classified, the predictive accuracy for GSE40419 and GSE50760 were 97.14% and 93.33%, respectively. GSE5364 includes 341 samples from multiple solid cancers: 18 lung tumor samples, 12 lung normal samples, 183 breast tumor samples, 13 breast normal samples, 9 colon tumor samples, 9 colon normal samples, 9 liver tumor samples, 8 liver normal samples, 16 oesophagus tumor samples, 13 oesophagus normal samples, 35 thyroid tumor samples, and 16 thyroid normal samples. LOOCV was carried out for tumor and normal samples of each tumor type in this data set, the predictive accuracy for lung, breast, colon, liver, oesophagus, and thyroid samples were 100%, 93.37%, 100%, 100%, 94.12%, and 68.63%, respectively. These results show that our 14-gene cross-cancer signature precisely differentiated between tumor and normal samples for all tumor types in GSE5364 except for those from the thyroid. Interestingly, we here were not able to effectively distinguish tumors from normal samples from the thyroid using this 14-gene cross-cancer signature, and this is consistent with the results from the TCGA data.

We found that CLUSTER1520 is a lung cancer-specific gene signature. In the 548 adjacent normal tissue samples of 12 TCGA cancer types, the expression level of CLUSTER1520 in the lung tissue samples was strikingly higher than any other tissue samples, and the same holds true for tumor samples if excluding THCA tumor samples from the analysis (Fig. 3). Moreover, CLUSTER1520 showed a substantially reduced level of expression in the lung tumor samples as compared to lung normal samples. In order to test if this signature can be used to differentiate lung tumors from other tumors, we divided all cancer samples from 12 TCGA cancer types into two classes: lung cancer samples (LUAD, LUSC) and non-lung cancer samples (BLCA, BRCA, COAD, HNSC, LIHC, KICH, KIRC, KIRP, PRAD, THCA), and performed LOOCV on these two classes of cancer samples using the expression values of CLUSTER1520. The predictive accuracy was 95.68%, namely we very effectively identified lung cancer samples out of a selection of 12 TCGA cancers based on the expression pattern of CLUSTER1520. We also validate that CLUSTER1520 is a lung cancer-specific gene signature on a non-TCGA microarray data set (GSE5364). GSE5364 includes 6 tumor types, and we divided those tumor samples into two classes: lung tumor samples and non-lung tumor samples (breast, colon, liver, oesophagus, thyroid). The predictive accuracy of LOOCV for these two classes of tumor samples was 100%, this demonstrated that lung tumor samples and non-lung tumor samples were accurately classified based on CLUSTER1520. These results show that CLUSTER1520 is a lung cancer-specific gene signature, and genes in this signature are potential targets for developing novel lung cancer therapies.

### Gene set association analysis of two non-TCGA data sets

In order to test if the significant gene sets we identified from TCGA data can be validated on non-TCGA data sources, we performed gene set association analysis on two non-TCGA cohorts: a lung adenocarcinoma data set (GSE40419) and a colorectal cancer data set (GSE50760), the results are shown in Supplementary Table S5. For GSE40419, we identified 1 significant gene set (FDR < 0.25), CLUSTER514, which is one of the seven cross-cancer gene signatures. Moreover, we found that 9 out of 10 top gene sets with smallest FDRs were within the significant gene sets identified from TCGA LUAD and/or LUSC data. For GSE50760, we identified five significant gene sets (FDR < 0.25), two of them, CLUSTER2556 and CLUSTER514, were also identified as significant using TCGA COAD data. In the 10 top gene sets with smallest FDRs, 4 overlap with the significant gene sets from TCGA COAD data. These results show that at least part of significant gene sets identified from TCGA data can be validated on these two non-TCGA data sets.

## Discussion

In this study, we comprehensively characterized the gene expression alterations of 12 types of cancers at the gene and gene set level. We identified DE genes and gene sets, some DE genes and gene sets are shared by different cancer types while others are only altered in one cancer type. We identified seven cross-cancer gene signatures that are differentially expressed in at least 4 cancer types. These signatures contain not only a number of genes which have established roles in cancers, but also genes that might be potential new biomarkers for cancers. These results reveal the aberrations in cancer transcriptomes and lead to a deeper understanding of the formation and development of human cancers.

Traditionally, we research one cancer type at a time, but there are patterns that can only be detected by making connections across different cancer types. Our results reveal that four gene sets, CLUSTER241, CLUSTER514, CLUSTER1011, and CLUSTER932, are significantly altered across seven cancer types: BLCA, BRCA, COAD, HNSC, LIHC, LUAD, and LUSC (Table 2). These similarities may indicate that there exist common mechanisms underlying the initiation and/or development of human cancers from different organs or different tissues in the same organ. Interestingly, three types of kidney tumors don’t show these patterns. We found that KIRC and KIRP are more similar to each other than KICH since they share 36% of DE genes (Table 1). Studies have shown that KICH is less aggressive than KIRC and KIRP^149^,^150^.

Gene expression changes with phenotypic consequences are driven by mutations and epimutations. The driver mutations and epimutations may be scattered in different pathways. We hypothesize that some of these mutations or epimutations may disrupt a pathway responsible for cell cycle regulation that directly drives cells into uncontrolled proliferation, while others may lie within an organ-specific pathway that turn a healthy cell into a cancer cell by altering the expression of cell cycle-associated pathways. We were not able to directly detect which pathways harbor the driver mutations through gene expression analysis, but we observed evidence that at least partially support this hypothesis: 1) aberrations in the cell cycle are a common feature shared by multiple cancer types since all of the cross-cancer gene signatures we identified involved cell cycle processes; 2) we found that some cancer-specific gene signatures contain genes implicated in corresponding organ-specific diseases; 3) each type of cancer has its unique features in terms of their DE profiles. It would be very interesting for future studies to explore the connections between mutational profiles and DE profiles and how gene expression patterns change surrounding driver mutations.

We identified some gene sets that were only significantly altered in one type of cancer. Some of these gene sets may be cancer-specific gene signatures, say CLUSTER1520 and CLUSTER2318, that shed light on the mechanisms underlying cancer-driving abnormalities in a specific organ, while many of them may still represent a cellular process broadly perturbed across cancer types, and the differentiation is just stronger in one cancer type than other cancer types. Therefore, when we look for mechanisms underlying a specific cancer type, we should treat these signatures with caution. It could be a way to reveal cancer-specific events by comparing various tumor types and looking into the differential gene sets between tumor types in the future.

A question arising from this study is how to make connections between the mutational profiles and DE profiles of human cancers. Some genes, for example tumor protein p53 (*TP53*/*p53*), phosphatidylinositol-4,5-bisphosphate 3-kinase, catalytic subunit alpha (*PIK3CA*), and retinoblastoma 1 (*RB1*), are frequently mutated in a number of cancers and are key genes contributing to tumorigenesis^151^,^152^,^153^. However, among 20530 genes in the BLCA, BRCA, COAD, HNSC, LIHC, LUAD, LUSC, KICH, KIRC, KIRP, PRAD, and THCA datasets, we found that *TP53* was ranked at 11834, 14601, 7752, 18515, 17359, 8769, 11116, 14995, 4200, 986, 4776, and 2094, *PIK3CA* at 16090, 7012, 14799, 4118, 13535, 13228, 6632, 12475, 14634, 17065, 19153, and 18438, *RB1* at 15691, 15157, 6836, 16116, 16543, 9168, 19208, 8333, 9565, 4233, 16756, and 11160, respectively (Supplementary Table S1). These genes are not at the top of the DE gene list. One reason could be that mutations in *TP53*/*PIK3CA*/*RB1* substantially change the expression of its downstream target genes rather than genes harboring them^154^. For example, the expression levels of two *p53* targets, *E2F7* and *HDGF*^69^,^130^, are significantly altered across multiple cancer types. These results indicate that cancer-causing genes may only have subtle expression changes, it is thus crucial to measure the total effect of a pathway or integrate mutation analysis into gene expression analysis.

Batch effects in high-throughput data might lead to inaccurate results when dealing with samples from multiple cancer types or data from different sequencing platforms^155^. In our study, all of the RNA-Seq data we used were from the same sequencing platform and same sequencing center, and this design minimizes the impact of batch effects on our analyses. Second, we clustered genes by their changing tendency in expression over samples, and this can eliminate the impact of batch effects on clustering since these effects are global effects on every gene in a sample. Third, we carried out gene set association analysis for each cancer type independently, thus avoiding the cross-cancer bias from the batch effects. Of course, proper handling of batch effects could improve the cross-platform or cross-cancer consistency when performing analyses on data from different sequencing platforms or different cancer types.

## Methods

### Overview

**Figure 6:**
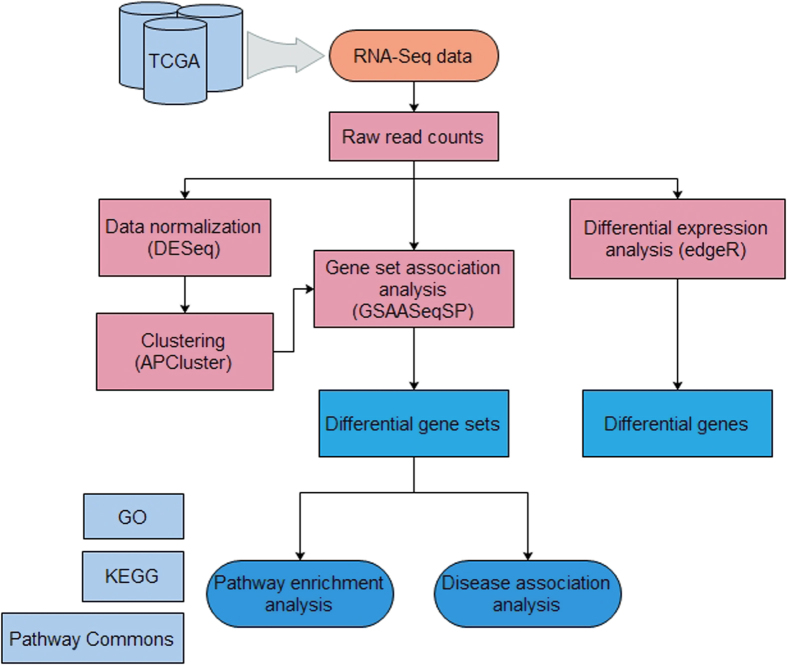
Pipeline of the analysis.

The pipeline of our analysis is illustrated in Fig. 6. The details of each step are described below.

### Data sets

Transcriptome data and clinical data were obtained from the TCGA Data Portal (https://tcga-data.nci.nih.gov/tcga/tcgaHome2.jsp). In order to eliminate the heterogeneity introduced by different sequencing platforms, we only downloaded those data in the category of UNC (IlluminaHiSeq_RNASeqV2). We chose 12 cancer types with transcriptome data available for both cancer and normal tissue samples. The two classes of phenotypes we used were “primary tumor” and “solid tissue normal”, namely only those samples in the clinical category of “primary tumor” or “solid tissue normal” were used for this study. The number of cancer and normal samples for each cancer type are listed in Table 4.

### Differential expression analysis of individual genes

Differential expression analysis of individual genes was carried out using the edgeR Bioconductor package^156^ (http://www.bioconductor.org/packages/release/bioc/html/edgeR.html). For each cancer type, we divide samples into two phenotypic groups, primary tumor and solid tissue normal, based on their clinical labels. Raw counts were extracted for these samples and edgeR was employed to find the differentially expressed genes between the two phenotypic groups.

### Clustering and gene set association analysis

In order to perform gene set association analysis, we first clustered genes into co-regulated sets based on their expression profiles over all normal samples across 12 cancer types. First, RNA-Seq data were normalized by the DESeq normalization method^157^, clustering was then performed using APCluster^158^,^159^ (http://www.bioinf.jku.at/software/apcluster/). We used the Pearson correlation coefficient to measure the similarity between genes. Pearson correlation measures the similarity in shape between two expression profiles, so this metric partitions genes into gene groups whose expression levels rise or fall synchronously under varying conditions or in response to a sequence of environment stimuli. We consider genes with coherent changing tendency in expression as co-regulated genes possibly functional in a same pathway. The number of clusters generated by APCluster is largely determined by the input preference, so we set the input preference (q) to 0.98 to obtain precise clusters, namely the expression profiles of genes in the same cluster are highly correlated.

We performed gene set association analysis for each cancer type to identify gene sets/clusters significantly associated with cancers. Gene set association analysis was carried out using GSAASeqSP, a software newly developed by our group^160^ (http://gsaa.unc.edu). RNA-Seq raw counts were normalized by the DESeq normalization in GSAASeqSP which is same as that in the DESeq Bioconductor package^157^. We chose Signal2Noise for gene-level differential expression analysis and Weighted_KS for gene set association analysis. Gene sets are gene clusters generated by APCluster, and one cluster represents one gene set. Gene sets with less than 15 genes or more than 100 genes were filtered to avoid overly narrow or broad functional categories. In this study, we set the FDR cutoff to 0.15, namely gene sets with FDR < 0.15 were considered to be statistically significantly associated with cancers.

### Pathway enrichment analysis and disease association analysis

To gain biological understanding of those gene sets statistically significantly associated with cancers, we carried out pathway enrichment analysis and disease association analysis using WebGestalt^161^,^162^ (http://bioinfo.vanderbilt.edu/webgestalt/). We conducted three types of pathway enrichment analyses for genes of significant gene sets: GO analysis, KEGG analysis, and Pathway Commons analysis. GO analysis is to find which GO terms are over-represented in a gene set based on the functional annotation of genes. KEGG analysis and Pathway Commons analysis are to discover pathways enriched in genes in a gene set, the difference between these two analyses is that KEGG analysis is based on the KEGG PATHWAY Database^163^ (http://www.genome.jp/kegg/pathway.html) while Pathway Commons analysis uses pathways collected by Pathway Commons^164^. Disease association analysis identifies diseases in which genes in a gene set are over-represented. We adopted the default values for parameters in WebGestalt when performing pathway enrichment analysis and disease association analysis.

## Additional Information

**How to cite this article**: Peng, L. *et al.* Large-scale RNA-Seq Transcriptome Analysis of 4043 Cancers and 548 Normal Tissue Controls across 12 TCGA Cancer Types. *Sci. Rep.* **5**, 13413; doi: 10.1038/srep13413 (2015).

## Supplementary Material

Supplementary Tables S1-S5

## Figures and Tables

**Table 1:**
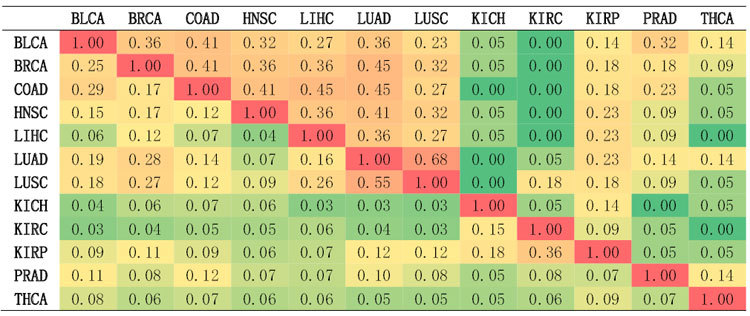
The percentage of common genes and gene sets in top 3% most differentially expressed genes and gene sets between 12 cancer types*.

*Results from DE gene comparisons lie below the diagonal line; results from DE gene set comparisons are above the diagonal line.

**Table 2:**
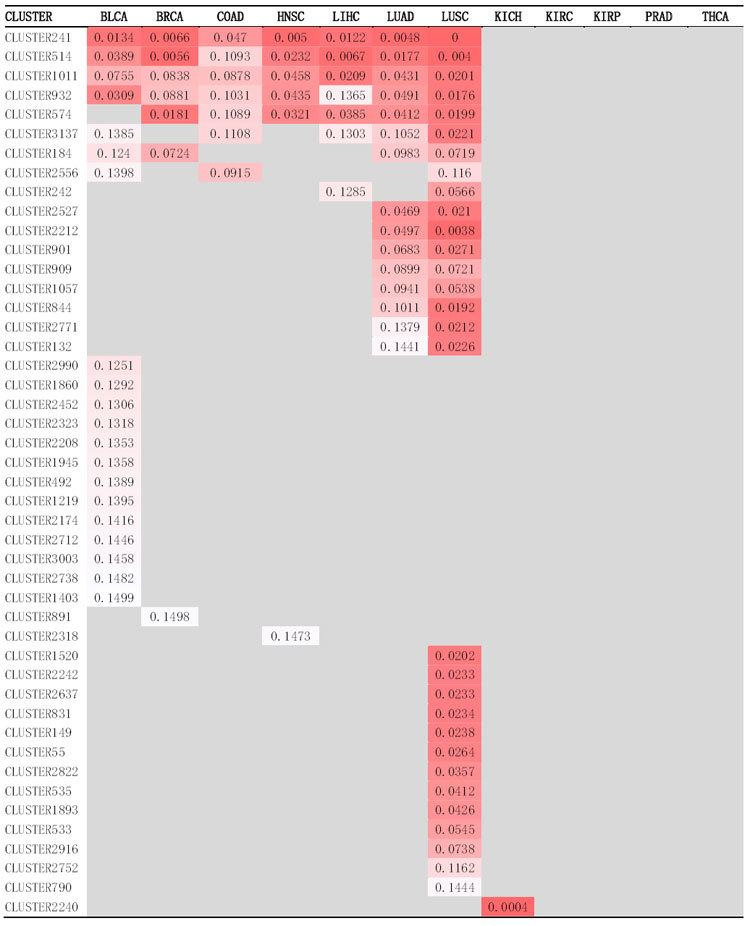
Significant gene sets at FDR < 0.15 from 12 types of cancers.

**Table 3:** List of lung disease-associated genes in CLUSTER1520.

Symbol	Full name	Lung diseases
SFTPA2	surfactant, pulmonary-associated protein A2	lung cancer^101^,^102^,^103^,^104^, acute and chronic lung disease^105^,^106^
SFTPA1	surfactant, pulmonary-associated protein A1	lung cancer^103^, acute and chronic lung disease^107^,^108^,^109^
ROS1	ROS proto-oncogene 1, receptor tyrosine kinase	lung cancer^165^,^166^,
ABCA3	ATP-binding cassette, sub-family A (ABC1), member 3	pulmonary fibrosis^167^, respiratory disease^168^,^169^,^170^
AQP4	aquaporin 4	lung cancer^171^,^172^
HHIP	hedgehog interacting protein	lung cancer^173^, chronic obstructive pulmonary disease^174^, abnormal lung function^175^
SLC34A2	solute carrier family 34, member 2	pulmonary alveolar microlithiasis^176^,^177^
IL6R	interleukin 6 receptor	chronic obstructive pulmonary disease^178^, abnormal lung function^179^

**Table 4:** Number of cancer and normal samples of 12 cancer types.

Abbreviation	Full Name	Number of Cancers	Number of Normals
BLCA	bladder urothelial carcinoma	185	16
BRCA	breast invasive carcinoma	955	106
COAD	colon adenocarcinoma	244	23
HNSC	head and neck squamous cell carcinoma	353	39
LIHC	liver hepatocellular carcinoma	123	46
LUAD	lung adenocarcinoma	450	58
LUSC	lung squamous cell carcinoma	398	44
KICH	kidney chromophobe	66	25
KIRC	kidney renal clear cell carcinoma	497	72
KIRP	kidney renal papillary cell carcinoma	127	28
PRAD	prostate adenocarcinoma	166	38
THCA	thyroid carcinoma	479	53
